# Ethanol Extract of *Alismatis rhizome* Inhibits Adipocyte Differentiation of OP9 Cells

**DOI:** 10.1155/2014/415097

**Published:** 2014-06-09

**Authors:** Yeon-Ju Park, Mi-Seong Kim, Ha-Rim Kim, Jeong-Mi Kim, Jin-Ki Hwang, Sei-Hoon Yang, Hye-Jung Kim, Dong-Sung Lee, Hyuncheol Oh, Youn-Chul Kim, Do-Gon Ryu, Young-Rae Lee, Kang-Beom Kwon

**Affiliations:** ^1^Center for Metabolic Function Regulation, Wonkwang University School of Medicine, 460 Iksan-daero, Iksan City, Jeonbuk 570−749, Republic of Korea; ^2^BK21 Plus program & Department of Smart Life-Care Convergence, Wonkwang University, Graduate School, 460 Iksan-daero, Iksan City, Jeonbuk 570−749, Republic of Korea; ^3^Department of Internal Medicine, Wonkwang University School of Medicine, 460 Iksan-daero, Iksan City, Jeonbuk 570−749, Republic of Korea; ^4^Department of Family Medicine, The Catholic University of Korea, Incheon St. Mary's Hospital, 56 Dongsu-ro, Bupyeong-gu, Incheon 403−720, Republic of Korea; ^5^Hanbang Body-Fluid Research Center, Wonkwang University, 460 Iksan-daero, Iksan City, Jeonbuk 570−749, Republic of Korea; ^6^Standardized Material Bank for New Botanical Drugs, College of Pharmacy, Wonkwang University, 460 Iksan-daero, Iksan City, Jeonbuk 570−749, Republic of Korea; ^7^Institute of Pharmaceutical Research and Development, College of Pharmacy, Wonkwang University, 460 Iksan-daero, Iksan City, Jeonbuk 570−749, Republic of Korea; ^8^Department of Korean Physiology, Wonkwang University School of Korean Medicine, 460 Iksan-daero, Iksan City, Jeonbuk 570−749, Republic of Korea; ^9^Department of Oral Biochemistry, and Institute of Biomaterials-Implant, Wonkwang University School of Dentistry, 460 Iksan-daero, Iksan City, Jeonbuk 570−749, Republic of Korea

## Abstract

The rhizome of *Alisma orientale (Alismatis rhizome)* has been used in Asia for promoting diuresis to eliminate dampness from the lower-*jiao* and to expel heat. In this study, an ethanol extract of the rhizome of *Alisma orientale* (AOE) was prepared and its effects on adipocyte differentiation of OP9 cells were investigated. Treatment with AOE in a differentiation medium for 5 days resulted in dose-dependent inhibition of lipid droplet formation in OP9 cells. Furthermore, AOE significantly inhibited adipocyte differentiation by downregulating the expression of the master transcription factor of adipogenesis, peroxisome proliferation-activity receptor **γ** (PPAR**γ**), and related genes, including CCAAT/enhancer binding protein **β** (C/EBP**β**), fatty acid-binding protein (aP2), and fatty acid synthase (FAS). AOE exerted its inhibitory effects primarily during the early adipogenesis stage (days 1-2), at which time it also exerted dose-dependent inhibition of the expression of C/EBP**β**, a protein related to the inhibition of mitotic clonal expansion. Additionally, AOE decreased the expression of autophagy-related proteins, including beclin 1, and the autophagy-related genes, (*Atg*) *7* and *Atg12*. Our results indicate that AOE's inhibitory effects on adipocyte differentiation of OP9 cells are mediated by reduced C/EBP**β** expression, causing inhibition of mitotic clonal expansion and autophagy.

## 1. Introduction

*Alismatis rhizome* (AR) is the rhizome of* Alisma orientale* (Sam.) Juzepzuk and belongs to the Alismataceae family. AR has been commonly used for the treatment of dampness-retention syndromes, such as edema, dysuria, and diarrhea in Asia. Furthermore, a number of experimental studies have reported its therapeutic potential as an anti-inflammatory [1, 2], antiallergic [3, 4], antibacterial [5], and antioxidant [6] agent. However, the effects of AR on adipocyte differentiation have not yet been investigated.

Obesity is a serious health problem and is related to the development of diseases such as type 2 diabetes, dyslipidemias, atherosclerosis, and even some cancers [7–10]. At the cellular level, obesity is characterized by increases in the number and volume of adipocytes, the primary storage site for energy in animals and humans. Adipogenesis is the process by which undifferentiated preadipocytes are converted to fully differentiated adipocytes [11]. At the onset of adipocyte differentiation, the expression of CCAAT/enhancer binding protein *β* (C/EBP*β*) and C/EBP*δ* is induced and these transcription factors are thought to mediate the expression of peroxisome proliferator-activated receptor *γ* (PPAR*γ*) and C/EBP*α* [12–14]. The activation of C/EBP*α* and PPAR*γ* leads to terminal differentiation through their subsequent transactivation of adipocyte-marker genes, such as adiponectin, lipoprotein lipase (LPL), fatty acid-binding protein 2 (aP2), and fatty acid synthase (FAS), all of which are involved in lipid metabolism [15].

Autophagy is a major, evolutionarily conserved, cytoplasmic degradation pathway that has also been implicated in adipose tissue development [16–18]. The genes encoding the basic components of the autophagy machinery are named* Atg* (autophagy-related) genes, and autophagy-deficient MEFs (*atg*5^−/−^,* atg*7^−/−^) exhibit markedly reduced efficiency during adipogenesis [16, 18].

Thus, in the present study, we set out to examine the antiadipogenic actions of an extract of* Alisma orientale* (AOE) rhizomes and to study the mechanisms involved.

## 2. Materials and Methods

### 2.1. Reagents

OP9 cells were purchased from the American Type Culture Collection (Manassas, VA, USA). Minimum essential medium alpha (MEM*α*), fetal bovine serum (FBS), Alexa Fluor 568 goat anti-rabbit IgG, and BODIPY 493/503 dye were purchased from Invitrogen (Carlsbad, CA, USA). Insulin, 3-isobutyl-1-methylxanthine (IBMX), dexamethasone (DEXA), and Oil Red O dye were purchased from Sigma Chemical Co. (St Louis, MO, USA). Antibodies against PPAR*γ*, C/EBP*α*, C/EBP*β*, cyclin A, cyclin D1, cyclin D2, beclin 1, ATG7, ATG13, LC3, insulin R *β*, and *β*-actin were purchased from Santa Cruz Biotechnology (Santa Cruz, CA, USA). Antibodies against extracellular signal-regulated kinases 1 and 2 (ERK1/2), phospho-ERK1/2, protein kinase B (Akt), and phospho-Akt were obtained from Cell Signaling Technology (Beverly, MA, USA). All of the chemicals used were of analytical grade.

### 2.2. Preparation of Extracts

Rhizomes of* Alisma orientale* (Alismataceae) were purchased in February 2010 from the University Oriental Herbal Drugstore, Iksan, Korea, and were identified by Professor Youn-Chul Kim, College of Pharmacy, Wonkwang University (Korea). A voucher specimen (Number WP10-02-4) was deposited at the Herbarium of the College of Pharmacy, Wonkwang University. Dried and pulverized rhizomes of* Alisma orientale* (50 g) were extracted twice with hot 70% ethanol (1 L) for 2 h at room temperature and filtered with filter paper. The filtrate was evaporated in* vacuo* to produce a 70% ethanol extract (12.15 g, 24.3 w/w%). The 70% ethanol extract was suspended in distilled water (100 mL), followed by filtration. The residue derived from the filtration was dissolved in hot ethanol and filtered again. The filtrate was then evaporated in* vacuo* to obtain a standardized fraction of* Alisma orientale* extract (AOE; NNMBS073, 600 mg, 1.2 w/w%). A 50 mg of AOE powder was dissolved in 1 mL of DMSO for treatment with OP9 cells. NNMBS073 was deposited at the Standardized Material Bank for New Botanical Drugs, Wonkwang University.

### 2.3. Cell Culture and Induction of Adipocyte Differentiation

OP9 cells were cultured in MEM*α*, containing 20% FBS, 2 mM l-glutamine, 100 U/mL penicillin, and 100 *μ*g/mL streptomycin, at 37°C in a 5% CO_2_ incubator. To induce differentiation, 1-day postconfluent preadipocytes were incubated in a differentiation medium, containing 10% FBS, 0.5 mM IBMX, 0.25 *μ*M DEXA, 175 nM insulin, 2 mM l-glutamine, 100 U/mL penicillin, and 100 *μ*g/mL streptomycin for 2 days. The medium was then changed to MEM*α*, containing 10% FBS, 2 mM l-glutamine, and 175 nM insulin, and the cells were cultured for a further 3 days.

### 2.4. Determination of Cell Viability

Effects of AOE on OP9 cell viability were determined using an established MTT assay. Briefly, cells were seeded in a 96-well dish and incubated at 37°C for 24 h to allow attachment. The attached cells were kept untreated or treated with 10, 20, and 40 *μ*g/mL AOE for various time periods at 37°C. The cells were then washed with phosphate-buffered saline (PBS) prior to adding MTT (0.5 mg/mL PBS) and incubating at 37°C for 30 min. Formazan crystals were dissolved with dimethyl sulfoxide (100 *μ*L/well) and detected at OD_570_ using an Emax Endpoint ELISA microplate reader (Molecular Devices, Sunnyvale, CA, USA).

### 2.5. Oil Red O Staining

After the induction of adipocyte differentiation, cells were washed with cold PBS, fixed at room temperature with 4% formalin for 1 h, and then rinsed with 60% isopropanol. OP9 cells were stained at room temperature with Oil Red O for 1 h and washed 4 times with distilled water. Intracellular Oil Red O dye was quantified by elution into isopropanol and measuring the OD_500_.

### 2.6. Automated Image Acquisition and Processing

After differentiation, adipocytes were washed with cold PBS, fixed at room temperature with 4% paraformaldehyde for 30 min, and then washed again 3 times with cold PBS. Blocking buffer was then added and incubated for 45 min at room temperature to prevent nonspecific antibody binding. PPAR*γ* or C/EBP*β* antibodies were added and incubated overnight. Cells were then washed 3 times and incubated for 1 h with BODIPY 493/503 dye for lipid droplet staining, DAPI for nuclear staining, and Alexa Fluor 568 goat anti-rabbit or anti-mouse IgG for PPAR*γ* and C/EBP*β*, respectively. Images were acquired on an ArrayScanTM VTi automated microscopy and image analysis system (Cellomics Inc., Pittsburgh, PA, USA). Using this automated and highly sensitive fluorescence-imaging microscope with a 20x objective and suitable filter sets, the stained cells were identified with DAPI in fluorescence channel 1, BODIPY 493/503 in channel 2, and Alexa Fluor 568 in channel 3, respectively. Arbitrary values for BODIPY, C/EBP*β*, and PPAR*γ* fluorescence were calculated from the standard deviation of pixel intensity under the DAPI channel, reflecting the intact DNA content of the cell.

### 2.7. Quantitative Real-Time Polymerase Chain Reaction (PCR)

Total RNA was extracted from cells using a FastPure RNA kit (TaKaRa, Shiga, Japan). The RNA concentration and purity were determined by absorbance at 260/280 nm. cDNA was synthesized from 1 *μ*g of total RNA using a PrimeScript RT reagent kit (TaKaRa). Expression of mRNA related to adipocyte differentiation was determined by real-time PCR, using the ABI PRISM 7900 Sequence Detection System and SYBR Green I (Applied Biosystems, Foster City, CA, USA). The primer sequences used are listed in Table 1. All results were normalized to the GAPDH housekeeping gene to control for variation in mRNA concentrations. Relative quantification was performed using the comparative ΔΔ*C*_*t*_ method according to the manufacturer's instructions.

### 2.8. Western Blot Analysis

OP9 cells were pretreated with 40 *μ*g/mL AOE for 1 h and then differentiated at 37°C. Cells were lysed with ice-cold mammalian protein extraction reagent (M-PER) (Pierce Biotechnology, Rockford, IL, USA), and the protein concentration in the lysate was determined using the Bradford method. Samples (20 *μ*g) were separated by sodium dodecyl sulfate-polyacrylamide gel electrophoresis with 10% acrylamide and transferred to Hybond-P polyvinylidene fluoride membranes (GE Healthcare Life Sciences, Buckinghamshire, UK) using a western blot apparatus. Each membrane was blocked for 2 h with 2% bovine serum albumin or 5% skim milk and then incubated overnight at 4°C with 1 *μ*g/mL of a 1 : 2,000 dilution of primary antibody. HRP-conjugated IgG (1 : 2,000 dilution) was used as the secondary antibody. Protein expression levels were determined by signal analysis using an image analyzer (Fuji-Film, Tokyo, Japan).

### 2.9. Statistical Analysis

Statistical analyses were performed using analysis of variance with Duncan's posttest; *P* values of >0.05 were considered to be statistically significant.

## 3. Results

### 3.1. Effects of AOE on Adipocyte Differentiation

**Figure 1:**
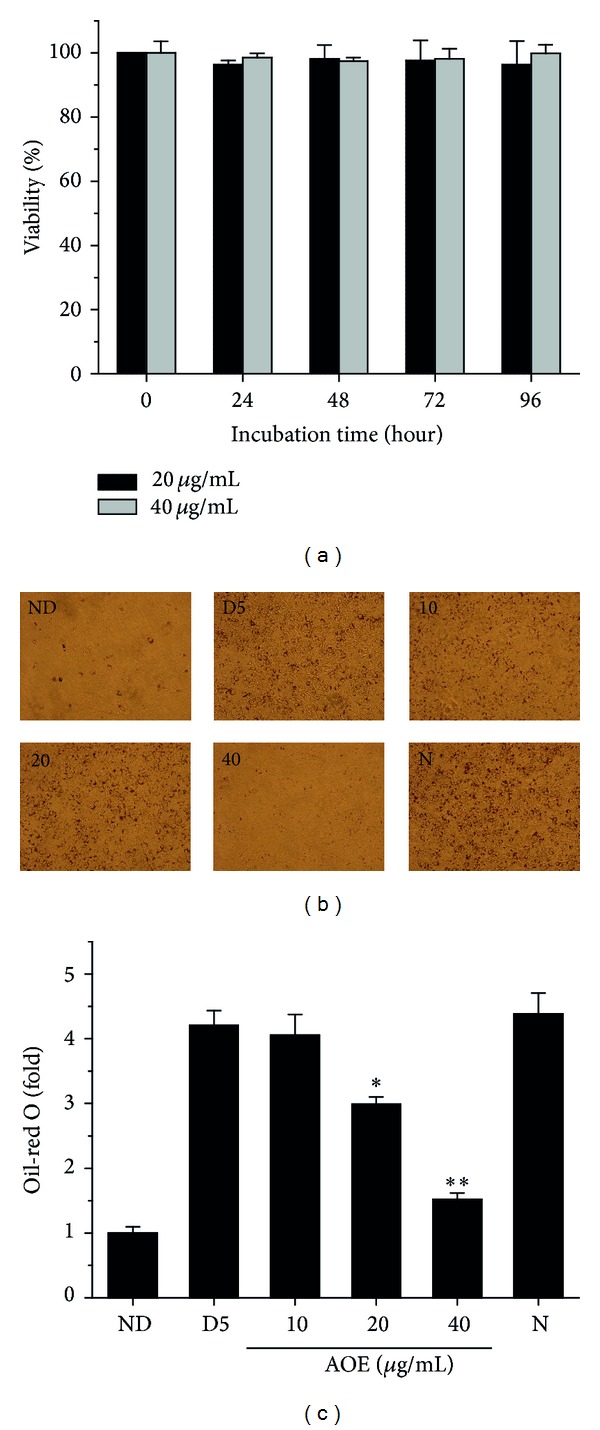
Effects of AOE on viability and lipid accumulation in OP9 cells. (a) Confluent OP9 cells were differentiated into adipocytes in medium containing adipogenic inducers for varying time intervals in the presence or absence of AOE (20 and 40 *μ*g/mL), as described in Materials and Methods. Effects of AOE on cell viability were measured by MTT assay and data were represented as relative cell viability values. ((b) and (c)) Cells were treated with adipogenic inducers to trigger differentiation into adipocytes in the presence or absence of various concentrations of AOE. After 5 days of differentiation, the cells were subjected to Oil Red O staining for a qualitative (b) and quantitative (c) comparison of intracellular lipid accumulation. The bars represent fold increases compared with ND groups. Data are presented as mean ± SD values of at least 3 independent experiments. *P < 0.05; **P < 0.01 compared to the D5 group. ND: no differentiation, D5: differentiation day 5, and N: negative control (20 *μ*g/mL Radix Astragali extract).

To investigate the actions of AOE on adipocyte differentiation, OP9 preadipocytes were induced to differentiate between the absence or presence of various concentrations of the extract. We observed that there were decreasing effects in lipid accumulation, as revealed by Oil Red O staining (Figures 1(b) and 1(c)). Following on from these results, we checked whether AOE might affect cell viability. We found that in differentiating cells treated for various periods with 20 or 40 *μ*g/mL AOE, cytotoxicity was not apparent when compared to the control cells (Figure 1(a)).

**Figure 2:**
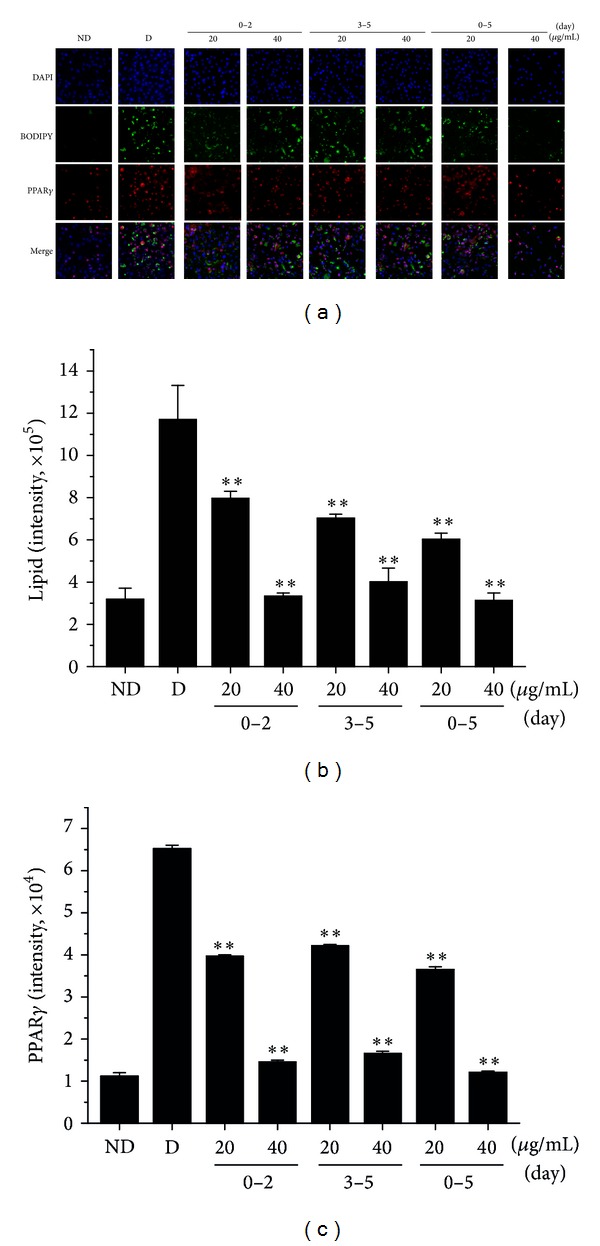
Effects of AOE on lipid droplet formation and PPAR*γ* expression in OP9 cells. OP9 cells were treated with adipogenic inducers to trigger differentiation into adipocytes. AOE 20 (40 *μ*g/mL) was then added during the early stage (0–2 days), late stage (3–5 days), or the entire period of differentiation (0–5 days). (a) After 5 days of differentiation, immunohistochemical staining of OP9 cells was carried out using a specific antibody to visualize PPAR*γ* (red), BODIPY 493/503 for lipid droplets (green), and DAPI to visualize nuclei (blue). (b) Total cellular lipid droplet content was determined by averaging cytosolic BODIPY intensities of individual cells. (c) PPAR*γ* levels were determined by averaging the intensities of nuclear antibody staining in individual cells. Approximately 5,000 cells were analyzed for lipid droplet content and PPAR*γ* levels. Data are expressed mean ± SD values of at least 3 independent experiments. **P < 0.01 compared to the D group. ND: no differentiation, D: differentiation.

To investigate at which stage of the differentiation process AOE inhibits adipogenesis, we treated the cells with AOE at different times, during the early (days 0–2) or late stages (days 3–5), or for the entire (days 0–5) differentiation period. The formation of lipid droplets and accumulation of triglyceride in adipocytes were blocked in all treatment groups, as revealed by BODIPY staining (green), which is specific for intracellular lipids (Figures 2(a) and 2(b)). We also stained PPAR*γ* protein to determine the expression level (red) and found it to be downregulated in all AOE-treated groups (Figures 2(a) and 2(c)).

### 3.2. Effects of AOE on Adipocyte Differentiation-Related Genes

**Figure 3:**
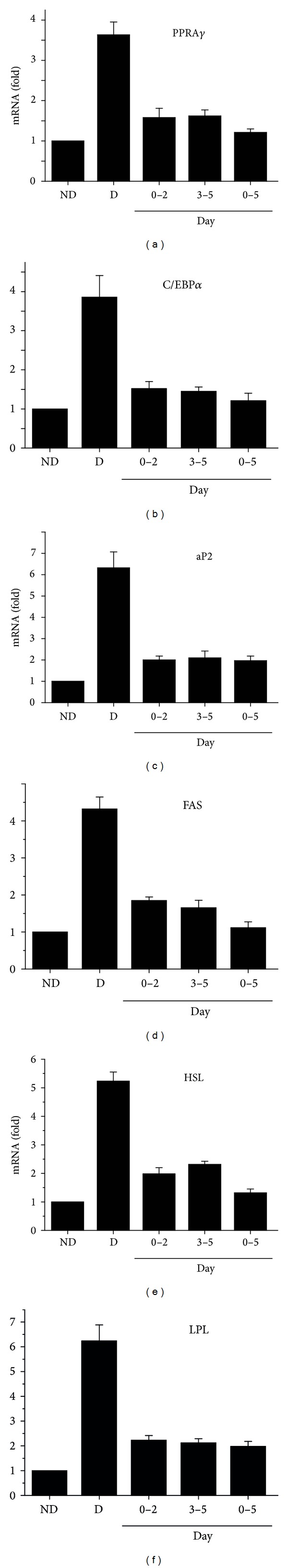
Effects of AOE on expression of PPAR*γ* and PPAR*γ*-target genes. OP9 cells were treated with adipogenic inducers to trigger differentiation into adipocytes. AOE (40 *μ*g/mL) was added during the early stage (0–2 days), late stage (3–5 days), or for the entire differentiation period (0–5 days). After 5 days of differentiation, real-time PCR was carried out using specific primers for PPAR*γ*, C/EBP*α*, aP2, FAS, HSL, and LPL, as described in Table 1. The genes transcript levels are expressed as ratios relative GAPDH expression, with the level in ND to 1. Data are expressed as mean ± SD values of at least 3 independent experiments. ND: no differentiation, D: differentiation.

Adipocyte differentiation is accompanied by increased expression of various transcription factors and adipocyte-specific genes. PPAR*γ* and C/EBP*α* are essential for terminal adipocyte differentiation and we found their expression to be significantly decreased in all treatment groups upon treatment with 40 *μ*g/mL AOE (Figure 3). We further investigated whether AOE-induced reduction of PPAR*γ* and C/EBP*α* levels regulated the expression of their target genes, including adipocyte protein 2 (aP2), fatty acid synthase (FAS), hormone-sensitive lipase (HSL), and lipoprotein lipase (LPL). Treatment with AOE (40 *μ*g/mL) significantly decreased expression of each of these proteins at various time intervals (Figure 3).

### 3.3. Effects of AOE on C/EBP*β* Expression during the Early Stage of Adipogenesis

**Figure 4:**
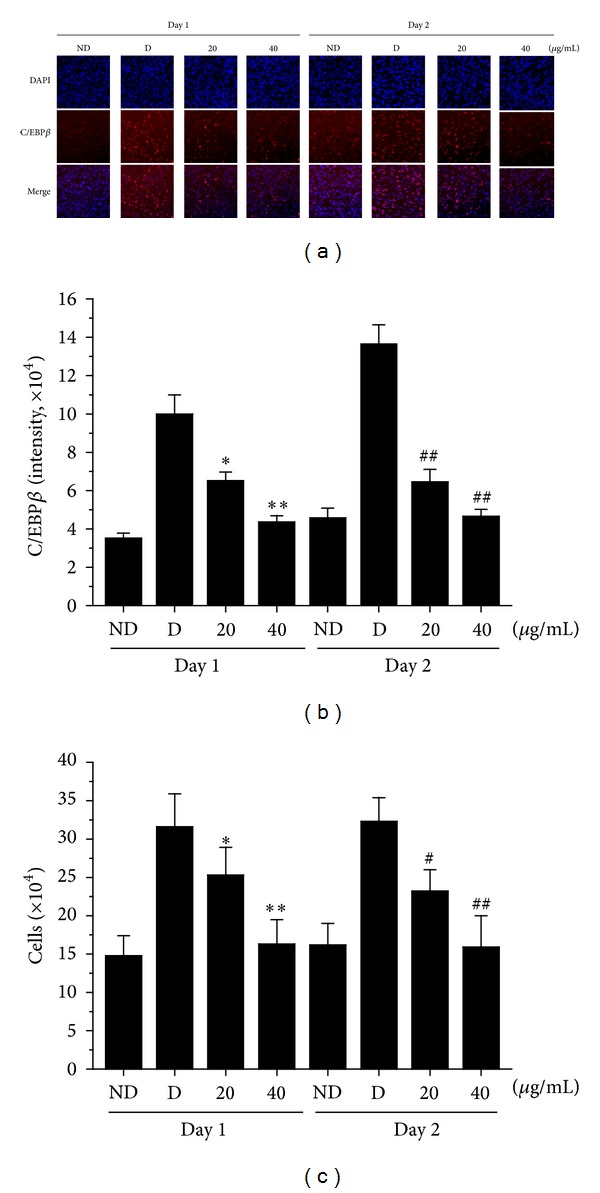
Effects of AOE on C/EBP*β* expression and cell proliferation in OP9 cells. (a) OP9 cells were pretreated with 20 or 40 *μ*g/mL AOE for 1 h and then cultured with adipogenic inducers. After 1 day and 2 days of differentiation, immunohistochemical staining of OP9 cells was carried out using a specific antibody to visualize C/EBP*β* (red) and DAPI to visualize nuclei (blue). (b) C/EBP*β* expression levels were determined by averaging the nuclear antibody staining intensity from 5,000 individual cells. (c) The number of cells treated with 20 and 40 *μ*g/mL AOE was determined using a hemocytometer. Experiments were carried out in triplicate and data are expressed as the mean ± SD values of at least 3 independent experiments. ^∗, #^ *P* < 0.05; ^∗∗, ##^ *P* < 0.01 compared to the D group at days 1 and 2, respectively. ND: no differentiation, D: differentiation.

C/EBP*β* is induced at a very early stage of adipogenesis and plays a crucial role in initiating the differentiation program by activating the expression of PPAR*γ* and C/EBP*α*, two key adipogenic transcription factors. We found that C/EBP*β* expression was significantly decreased during the early stage of adipogenesis (days 0–2) in OP9 adipocytes treated with either 20 or 40 *μ*g/mL of AOE (Figures 4(a) and 4(b)). When growth-arrested preadipocytes were treated with adipogenic inducers during the early stage, the number of adipocytes increased by approximately twofold. This response was significantly inhibited by treatment with AOE, with the number of AOE-treated OP9 adipocytes being similar to that of the control group (Figure 4(c)).

### 3.4. Effects of AOE on Cyclin and Autophagy-Related Protein Expression

**Figure 5:**
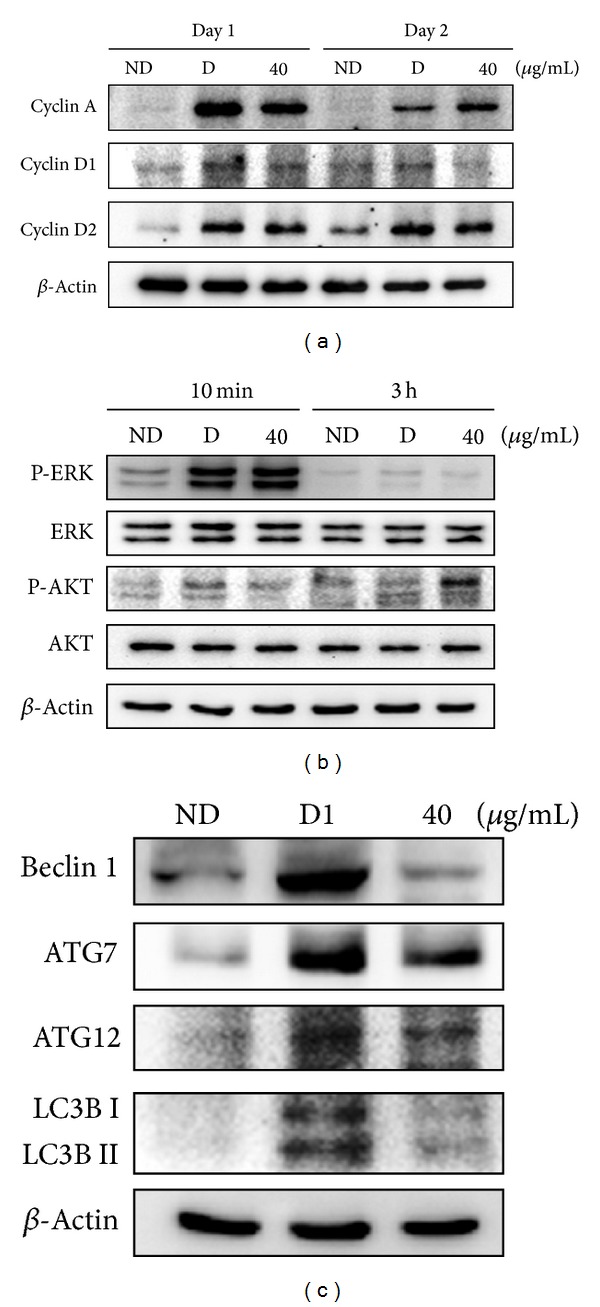
Effects of AOE on cell proliferation and autophagy-related protein expression in OP9 cells. OP9 cells pretreated with 40 *μ*g/mL AOE were then cultured with adipogenic inducers for the designated times. After harvesting, the lysates were subjected to western blot analysis for cyclins A, D1, and D2 (a) and ERK, p-ERK, Akt, and p-Akt (b) and beclin1, Atg7, Atg12, and LC3B I and II (c). ND: no differentiation, D: differentiation, D1: differentiation day 1.

To determine the signaling pathway through which AOE inhibits clonal expansion during the early stage of adipogenesis, cyclins A, D1, and D2, ERK, and Akt protein expressions were examined. As shown in Figures 5(a) and 5(b), cyclin D1 expression was decreased, while cyclins A and D2 were not altered by treatment with 40 *μ*g/mL AOE. Furthermore, ERK and AKT phosphorylations were both increased after treatment with adipogenic inducers for 10 min, but treatment with AOE attenuated AKT, but not ERK, phosphorylation. Since autophagy is one of the major factors involved in regulating the early events in adipocyte differentiation, we also examined the expression of autophagy-related proteins after 1 day of treatment with AOE. We found beclin1, Atg7, Atg12, and LC3 expression to be increased after 1 day of treatment with adipogenic inducers. However, in cells treated with 40 *μ*g/mL AOE, activation of autophagy was significantly inhibited, as indicated by reduced levels of beclin1, Atg7, Atg12, and LC3 proteins (Figure 5(c)).

## 4. Discussion and Conclusions

In the present study, we show that AOE exerts anti-adipogenic effects by a complex mechanism. We found that AOE treatment remarkably reduced levels of Oil Red O staining in a concentration-dependent manner, without affecting cell viability (Figure 1).

It is well known that when preadipocytes are induced to differentiate, the cells first initiate several rounds of mitotic clonal expansion and then become quiescent while the coordinated transcription of adipogenic genes is initiated [19]. Preadipocytes undergo mitotic clonal expansion through upregulation of C/EBP*β* and C/EBP*δ* during the early stage of adipocyte differentiation (day 0–2) [13]. In this study, we found that, during the early stage, AOE inhibited lipid droplet formation and suppressed C/EBP*β* expression. AOE also inhibited adipocyte differentiation through suppression of cell proliferation. Cell proliferation during adipogenesis occurs through the G_1_/S checkpoint, as shown by activation of CDK2-cyclin E/A and cyclin D1, turnover of P27^kip1^, and hyperphosphorylation of Rb protein [20]. Furthermore, the PI3K/Akt pathway affects cell cycle progression, through regulation of cyclin D and P27^kip1^ expression [21]. Here, we found that AOE treatment decreased cyclin D expression and phosphorylation of Akt in response to adipogenic inducers in OP9 cells (Figures 5(a) and 5(b)). In contrast, while adipogenic inducers also stimulate the MEK/ERK pathway, resulting in enhanced activity of C/EBP*β* and induction of adipocyte differentiation [22], AOE-treatment does not alter this response. These results suggest that AOE may exert its inhibitory effects on adipocyte differentiation via downregulation of cyclin D1 and C/EBP*β* expression, which, in turn, happens because of decreased Akt activation.

PPAR*γ* and C/EBP*α* are both known to be direct transcriptional activators of adipocyte differentiation, and the most characterized adipocyte-specific regulatory DNA motifs contain binding sites for both factors [23]. PPAR*γ* is known to bind to the promoter region that induces the expression of C/EBP*α*, which is, in turn, regulated by C/EBP*β* during adipocyte differentiation [24]. C/EBP*β* is expressed at earlier time points than both C/EBP*α* and PPAR*γ* during adipogenesis [22], and it is known to induce the expression of C/EBP*α* and PPAR*γ* [15]. We found that AOE considerably reduces levels of C/EBP*β* protein during the early phase (Figures 4(a) and 4(b)), and that it also significantly inhibits expression of C/EBP*α* and PPAR*γ* at the mRNA level (Figure 3). It has been reported that adipogenesis-related genes, such as aP2, FAS, HSL, and LPL, are targets of PPAR*γ* and C/EBP*α*. In our study, AOE inhibited the expression of each of these adipocyte-specific genes (Figure 3), suggesting that AOE inhibits the expression of C/EBP*β* during the early stage of adipogenesis. This, in turn, leads to reduced expression of PPAR*γ* and C/EBP*α*, ultimately leading to inhibition of lipid accumulation.

A recent advance in understanding adipogenesis is the role played by autophagy, a catabolic process for degradation of bulk cytoplasmic contents and subcellular organelles [25]. Most of the genes involved in autophagy, named autophagy-related genes (Atg), have been identified. Importantly, genetic deletion of Atg5 and Atg7, two essential autophagy genes, significantly inhibits adipocyte differentiation in 3T3-L1 cells and attenuates diet-induced obesity in mice [16, 18]. We found that after 1 day of differentiation, levels of autophagy-related specific proteins in OP9 cells, such as beclin 1, Atg7, Atg12, and LC3, were increased by adipogenic inducers. However, in the AOE-treated groups, increases in autophagy-related proteins were repressed (Figure 5(c)). These findings demonstrate the functional importance of autophagy inhibition in AOE-induced repression of adipocyte differentiation in OP9 cells. Since early induction of both C/EBP*β* expression and autophagy is crucial for adipocyte differentiation, it is plausible that the inhibition of autophagy induced by AOE may be related to the decrease of C/EBP*β* expression.

In summary, we have shown that AOE suppresses adipocyte differentiation in OP9 cells by downregulating the expression of C/EBP*β* and consequently decreasing PPAR*γ* and C/EBP*β* levels. AOE also inhibits adipogenesis by attenuating autophagy in response to adipogenic inducers. Thus, our studies show that AOE exerts antiadipogenic actions and therefore has promising therapeutic potential in preventing obesity.

## Figures and Tables

**Table 1:** Primers and probes for real-time quantitative PCR.

Genes	Primer sequence	Accession no.
PPAR*γ*	5′-GAAAGACAACGGACAAATCACC-3′ 5′-GGGGGTGATATGTTTGAACTTG-3′	NM_011146

C/EBP*α*	5′-TTGTTTGGCTTTATCTCGGC-3′ 5′-CCAAGAAGTCGGTGGACAAG-3′	NM_007678

FABP4	5′-AGCCTTTCTCACCTGGAAGA-3′ 5′-TTGTGGCAAAGCCCACTC-3′	NM_024406

FAS	5′-TGATGTGGAACACAGCAAGG-3′ 5′-GGCTGTGGTGACTCTTAGTGATAA-3′	NM_007988

HSL	5′-GGAGCACTACAAACGCAACGA-3′ 5′-TCGGCCACCGGTAAAGAG-3′	NM_010719

LPL	5′-GGACGGTAACGGGAATGTATGA-3′ 5′-TGACATTGGAGTCAGGTTCTCTCT-3′	NM_008509

GAPDH	5′-CGTCCCGTAGACAAAATGGT-3′ 5′-TTGATGGCAACAATCTCCAC-3′	NM_008084

## References

[1] Bravo L (1998). Polyphenols: chemistry, dietary sources, metabolism, and nutritional significance. *Nutrition Reviews*.

[2] Dai Y, Hang B, Huang Z, Li P (1991). Anti-inflammatory activities and effect of rhizoma Alismatis on immune system. *Zhongguo Zhong Yao Za Zhi*.

[3] Lee JH, Kwon OS, Jin H-G, Woo E-R, Kim YS, Kim HP (2012). The rhizomes of Alisma orientale and alisol derivatives inhibit allergic response and experimental atopic dermatitis. *Biological and Pharmaceutical Bulletin*.

[4] Matsuda H, Kageura T, Toguchida I, Murakami T, Kishi A, Yoshikawa M (1999). Effects of sesquiterpenes and triterpenes from the rhizome of Alisma orientale on nitric oxide production in lipopolysaccharide-activated macrophages: absolute stereostructures of alismaketones-B 23-acetate and -C 23-acetate. *Bioorganic and Medicinal Chemistry Letters*.

[5] Jin H-G, Jin Q, Ryun Kim A (2012). A new triterpenoid from Alisma orientale and their antibacterial effect. *Archives of Pharmacal Research*.

[6] Han CW, Kang ES, Ham SA, Woo HJ, Lee JH, Seo HG (2012). Antioxidative effects of Alisma orientale extract in palmitate-induced cellular injury. *Pharmaceutical Biology*.

[7] Hursting SD, Lashinger LM, Colbert LH (2007). Energy balance and carcinogenesis: underlying pathways and targets for intervention. *Current Cancer Drug Targets*.

[8] Lau DCW, Dhillon B, Yan H, Szmitko PE, Verma S (2005). Adipokines: molecular links between obesity and atheroslcerosis. *American Journal of Physiology: Heart and Circulatory Physiology*.

[9] Rajala MW, Scherer PE (2003). Minireview: the adipocyte—at the crossroads of energy homeostasis, inflammation, and atherosclerosis. *Endocrinology*.

[10] Zimmet P, Alberti KGMM, Shaw J (2001). Global and societal implications of the diabetes epidemic. *Nature*.

[11] Otto TC, Lane MD (2005). Adipose development: from stem cell to adipocyte. *Critical Reviews in Biochemistry and Molecular Biology*.

[12] Gregoire FM, Smas CM, Sul HS (1998). Understanding adipocyte differentiation. *Physiological Reviews*.

[13] Ntambi JM, Young-Cheul K (2000). Adipocyte differentiation and gene expression. *Journal of Nutrition*.

[14] Tong Q, Hotamisligil GS (2001). Molecular mechanisms of adipocyte differentiation. *Reviews in Endocrine and Metabolic Disorders*.

[15] Farmer SR (2006). Transcriptional control of adipocyte formation. *Cell Metabolism*.

[16] Baerga R, Zhang Y, Chen P-H, Goldman S, Jin S (2009). Targeted deletion of autophagy-related 5 (atg5) impairs adipogenesis in a cellular model and in mice. *Autophagy*.

[17] Tsukada M, Ohsumi Y (1993). Isolation and characterization of autophagy-defective mutants of Saccharomyces cerevisiae. *FEBS Letters*.

[18] Zhang Y, Goldman S, Baerga R, Zhao Y, Komatsu M, Jin S (2009). Adipose-specific deletion of autophagy-related gene 7 (atg7) in mice reveals a role in adipogenesis. *Proceedings of the National Academy of Sciences of the United States of America*.

[19] MacDougald OA, Lane MD (1995). Adipocyte differentiation. When precursors are also regulators. *Current Biology*.

[20] Choi K-M, Lee Y-S, Sin D-M (2012). Sulforaphane inhibits mitotic clonal expansion during adipogenesis through cell cycle arrest. *Obesity*.

[21] Muise-Helmericks RC, Grimes HL, Bellacosa A, Malstrom SE, Tsichlis PN, Rosen N (1998). Cyclin D expression is controlled post-transcriptionally via a phosphatidylinositol 3-kinase/Akt-dependent pathway. *Journal of Biological Chemistry*.

[22] Tang Q-Q, Otto TC, Daniel Lane M (2003). Mitotic clonal expansion: a synchronous process required for adipogenesis. *Proceedings of the National Academy of Sciences of the United States of America*.

[23] White UA, Stephens JM (2010). Transcriptional factors that promote formation of white adipose tissue. *Molecular and Cellular Endocrinology*.

[24] Hou Y, Xue P, Bai Y (2012). Nuclear factor erythroid-derived factor 2-related factor 2 regulates transcription of CCAAT/enhancer-binding protein *β* during adipogenesis. *Free Radical Biology and Medicine*.

[25] Levine B, Yuan J (2005). Autophagy in cell death: an innocent convict?. *Journal of Clinical Investigation*.

